# The Implication of Androgens in the Presence of Protein Kinase C to Repair Alzheimer’s Disease-Induced Cognitive Dysfunction

**DOI:** 10.29252/ibj.24.2.64

**Published:** 2019-11-01

**Authors:** Sara Amiri, Kayhan Azadmanesh, Marzieh Dehghan Shasaltaneh, Vafa Mayahi, Nasser Naghdi

**Affiliations:** 1 Department of Physiology and Pharmacology, Pasteur Institute of Iran, Tehran, Iran;; 2 Department of Virology, Pasteur Institute of Iran, Tehran, Iran;; 3 Department of Biology, Faculty of Science, University of Zanjan, Zanjan, Iran;; 4 Department of Microbiology, Islamic Azad University, Karaj, Iran

**Keywords:** Androgens, Cognition, Hippocampus, Protein kinase C, Spatial memory

## Abstract

Aging, as a major risk factor of memory deficiency, affects neural signaling pathways in hippocampus. In particular, age-dependent androgens deficiency causes cognitive impairments. Several enzymes like PKC are involved in memory deficiency. Indeed, PKC regulatory process mediates α-secretase activation to cleave APP in β-amyloid cascade and tau proteins phosphorylation mechanism. Androgens and cortisol regulate PKC signaling pathways, affecting the modulation of RACK1. Mitogen-activated protein kinase/ERK signaling pathway depends on CREB activity in hippocampal neurons and is involved in regulatory processes via PKC and androgens. Therefore, testosterone and PKC contribute in the neuronal apoptosis. The present review summarizes the current status of androgens, PKC, and their influence on cognitive learning. Inconsistencies in experimental investigations related to this fundamental correlation are also discussed, with emphasis on the mentioned contributors as the probable potent candidates for learning and memory improvement.

## INTRODUCTION

Memory is correlated with several factors, including time, space, and content. Indeed, memory process is mediated by molecular events, which affect neural signaling pathways^[^^1^^,^^2^^]^. The subsequent alterations in neural synapses occur in hippocampus and related cortices^[^^3^^]^. Thus, synaptic impairment leads to memory defects in hippocampus^[^^2^^]^. The involvement of subcortical structures including “hippocampus” in learning and memory processes has been well established in the mammalian brain. Various neuroactive steroids (e.g. androgens) receptors are also found in hippocampal CA1 pyramidal cells, which strengthens the fact that hippocampus is an important target for steroids and their neuromodulatory actions^[^^4^^,^^5^^]^. Steroids exert their impacts via genomic and non-genomic pathways. Two important androgens, DHT and DHEA, activate the enzymes engaged in the memory processes, especially all isoforms of PKC^[^^6^^]^. PKC is crucial for hippocampal memory formation and alterations in PKCγ contribute to deficits in hippocampal-mediated memory in the aged individuals^[^^7^^]^. PKC is involved in physiological processes related to learning and memory^[^^8^^,^^9^^]^ and is called cognitive kinase^[^^10^^]^. This enzyme regulates synaptic transmission. Furthermore, PKC and several of its substrates, including myristoylated alanine-rich C-kinase substrate, GAP-43, and NMDA receptor, are involved in the information processing and storage^[^^11^^-^^14^^]^. The PKC phosphorylation site plays a key role in regulating memory-associated tasks for GAP-43^[^^15^^]^. In addition to the efficacy of neural signaling pathways, memory process correlates with the number of synapses and their function. In fact, the dynamic feature of synapses and their action depend on their probable significant alteration in shape, density, and function in reaction to memory requirements^[^^16^^]^.

Aging can lead to deficiency in functional and behavioral processes such as memory. Two major cognitive functions are working (temporary) memory and declarative LTM including episodic or semantic learning. Working memory is related to prefrontal cortex, while declarative memory is associated with hippocampus, perirhinal, entorhinal, and para-hippocampal cortices. Deficiencies in working and declarative memory seem to be linked to aging^[^^17^^]^. Aged men’s and women’s cognitive learning show a functional decline and is mostly influenced by episodic memory dysfunction^[^^18^^]^. Attention and executive control are also found to be degenerated by aging, for which aged humans fail to switch their attention between several tasks^[^^19^^]^, or their ability to organize, plan, evaluate, or coordinate is impaired^[^^20^^]^. The age-dependent memory dysfunction also leads to the enhanced vulnerability of brain to injury and various types of dementia as a consequence^[^^21^^]^. Indeed, the integrity and efficiency of functional processes seem to be decreased in hippocampus throughout the lifetime^[^^22^^]^. This hypothesis has also been approved by subsequent studies, representing that execution of memory tasks related to younger rodents are performed more efficiently and more rapidly than aged ones^[^^23^^-^^25^^]^. Additionally, dentate gyrus region of hippocampus has shown a decreased rate of metabolism and volume in elderly humans, monkeys, and mice, which was connected to memory dysfunction^[^^26^^-^^29^^]^. Overall, memory disorders, including AD, vascular dementia, PD, dementia, Huntington’s disease, frontotemporal dementia, traumatic brain injury leading to memory impairments, mental retardation, depression, alcohol-related dementia, and Creutzfeldt–Jakob disease, are caused by deficiency in synaptic pathways, e.g. synaptic impairment and loss^[^^30^^]^. Furthermore, many of human cognitive processes are found to be sex-dependent, suggesting that females are more potent in verbal fluency, while spatial working memory is believed to be reinforced in males^[^^31^^-^^33^^]^. Cognitive impairments that target neurological and psychiatric diseases, e.g. PD and schizophrenia, also represent a sex-related nature, developing in men more frequently than women^[^^34^^-^^37^^]^. Moreover, AD with a sex-dependent feature has been characterized as the severe, progressive neuro-degenerative disorder, which is believed to be the cause of up to 80% of dementia in over 60-year-old individuals throughout the world, leading to memory impairment, cognitive dysfunctions, behavioral decline, loss of ability to learn, and consequently death^[^^38^^-^^40^^]^. More than 35 million individuals are affected by AD worldwide, for which its incidence rate appears to be increasing by aging^[^^40^^]^. In addition, studies have recommended that depletion of steroid hormones associated with aging may have major impacts on development of AD^[^^17^^]^. Pathogenesis study of AD has also demonstrated that this neuro-degenerative disorder is associated with two major pathological symptoms: extracellular amyloid plaques and intracellular neurofibrillary tangles (tau protein)^[^^41^^]^. 

**Fig 1:**
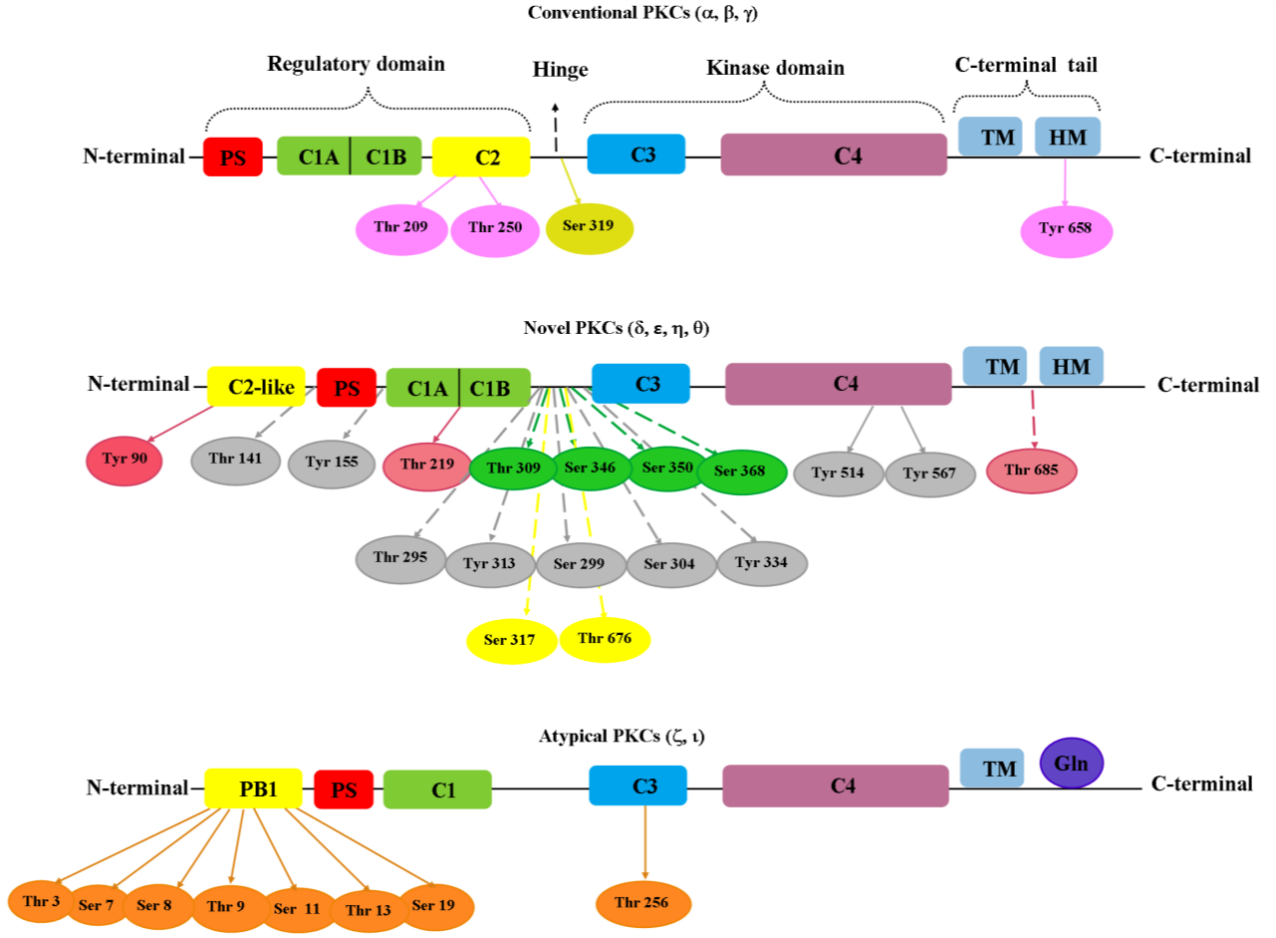
Phosphorylation sites identified on PKC isoforms. Phosphorylation sites of each isoform have been presented as a color oval. Purple color, conventional PKCα,  , and  ; bitter lemon color (Ser 319), conventional PKCα novel PKC; pink color, PKC ; gray color, PKC ; green color, PKC ε; yellow color, PKC  ; orange color, atypical PKCs. PS, pseudosubstrate; TM, turn motif; HM, hydrophobic motif; PB1, phosphatidylserine-binding domains; Gln, glutamine

Aβ performs a critical role in AD pathogenesis. Although Aβ leaves the neurons, it may be found in astrocytes and microglia^[^^42^^]^. Aggregation of insoluble Aβ plaques in brain is generated by APP cleavage, in addition to tau (MT protein) hyperphosphorylation, oxidative stress, and reactive glial, and microglial changes^[^^43^^,^^44^^]^. Despite the wide therapeutic strategies applied for AD patients in recent years, credible biomarkers are still needed for disease diagnosis at early stages. Moreover, the routinely used drugs only demonstrate effective impacts on disease’s later stages, with only half of the patients showing decreased levels of development pace for behavioral and cognitive symptoms, which only suggests delay in the process of symptoms progression, not significant inhibition or cure of the AD^[^^39^^,^^44^^]^. In the neurons of AD patients, the first abnormality is a defect in PKC signaling pathway. Inhibition of PKC activity leads to the reduced learning and memory capacity^[^^9^^]^. Based on the types of PKC isoforms, there are several phosphorylation sites^[^^45^^]^with central roles in regulating memory-associated tasks^[^^15^^] ^(Fig. 1). Activation of PKC inhibits the activity of GSK3; hence, hyperphosphorylation of tau protein is prevented, and finally the accumulation of Aβ peptide is reduced. This review will focus on PKC and its role in cognitive function associated with androgen hormone, suggesting a relation with other signaling pathways. 

**Androgens classification**

Hormones have a potent impact on several biological mechanisms during the life, while causing physiological alterations to specific tissues in major developmental periods. In animals, some hormones promote physiological behaviors or processes, which seem to be sex-dependent. Indeed, steroid hormones play a significant role, as these chemical messengers affect the structural and functional organization of various tissues in the body, which subsequently results in sexual differences^[^^46^^,^^47^^]^. The major types of steroid hormones include progestins, androgens, estrogens, and corticoids^[^^48^^,^^49^^]^. Male gonadal hormones, which are characterized as essential agents responsible for the development and maintenance of the male reproductive system, are known as androgens^[^^50^^,^^51^^]^. In fact, 5α-DHT is the most biologically active sex hormone produced by enzymatic conversion of testosterone via 5α-reductase. However, ARs mediate approximately every biological operation of endogenous androgens^[^^52^^]^.

**Genomic and non-genomic pathways of androgens**

**Fig 2:**
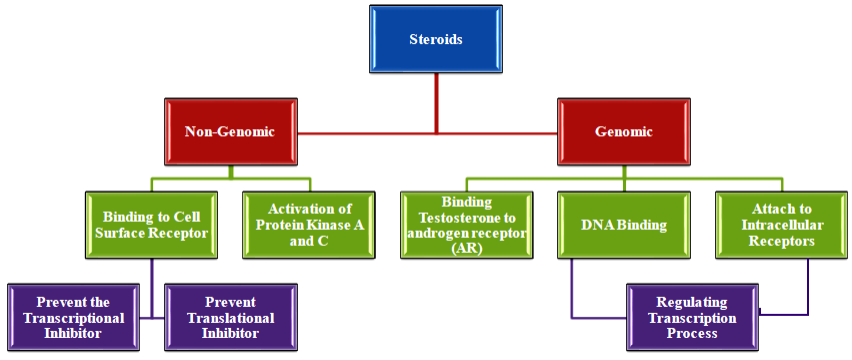
Role of steroids in the cell signaling pathways

Steroids exert their impacts via genomic and non-genomic pathways. In order to exert genomic influence, steroid hormones are known to attach to intracellular receptors and specific DNA sequences while regulating gene transcription. The non-genomic pathway is a rapid mechanism using cell surface receptors in brain and neuroendocrine systems^[^^53^^]^ with the ability to prevent the transcriptional and translational inhibitors^[^^54^^]^. Furthermore, non-genomic pathway of androgens is involved in the formation of second messengers and activation of PKA and PKC signaling pathways^[^^55^^]^. However, previous studies have shown that most androgens impacts are exerted through genomic pathway via AR^[^^56^^]^. Nonetheless, several investigations have demonstrated that both pathways are defective in terms of LTM^[^^57^^-^^60^^]^ (Fig. 2). The activation of PKC is dependent on G protein like Gq. Phospholipase C is typically activated via coupling with the G protein Gq and results in the DAG, a key allosteric activator of PKC. Isozymes of PKC signaling pathway in brain is regulated through DG^[^^61^^,^^62^^]^.

According to studies, hippocampus is known to be involved in spatial cognition and memory processes, and a site for the occurrence of neuromodulatory actions related to androgens, e.g. testosterone, which is mainly characterized to be a spatial learning determinant in male rats^[^^63^^-^^65^^]^.

Male major androgens are testosterone, DHT, androstenedione, and DHEA, as well as its sulfate derivatives (DHEA-S). Nevertheless, from the biological aspect the main androgen is testosterone, found in cerebral cortex and hippocampus, which attaches directly to AR or transforms to active DHT by 5α-reductase^[^^66^^]^. Furthermore, DHEA and DHEA-S functions in central nervous system are determined as neuroactive steroids due to their neuronal regulating activities^[^^17^^]^. Studies have revealed that rat CA1 pyramidal cells in hippocampus are the site of AR immunoreactivity, where androgens are able to increase male excitability of neurons. Moreover, the regulation of hippocampus-mediated behaviors are dependent on androgens^[^^63^^]^. Generally, androgens levels decrease in male over lifetime. The deficiency of androgens leads to muscle mass and strength decline, behavioral and emotional changes, depression, memory impairment, and cognitive dysfunction, which are associated with AD^[^^66^^]^. Concerning the involvement of androgens in cognition, a number of studies have been performed so far. Positive correlation of endogenous testosterone and spatial learning has previously been confirmed in healthy men^[^^65^^,^^67^^]^. Nonetheless, following many other researches, verification of positive connection of endogenous testosterone and spatial ability is rejected^[^^68^^-^^70^^]^. Such inconsistencies in results may be due to wide range of androgens functions or the detailed differences in experimental conditions.

Overall, due to the essential role of androgens in cognitive learning, androgen replacement therapy has become under focus. Previous findings have revealed that vascular dementia patients indicate memory improvement when treated with androgen replacement therapy^[^^71^^,^^72^^]^. Scientists have investigated testosterone levels in orchidectomized rats, and their results demonstrated that testosterone treatment using tenfold concentration levels greater than normal testosterone levels leads to memory improvement^[^^73^^]^. Hawley *et al*.^[^^64^^]^ have also confirmed the ideas of spatial cognition improvement resulted by testosterone therapy in rodents. Formerly, it has determined that LTM could be impaired in the presence of testosterone in passive avoidance conditioning both via intracellular AR and through the non-genomic effects of steroids^[^^74^^-^^76^^]^. DHEA-S activates an allosteric site on the GABA receptor that inhibits chloride channel opening, thus increasing neuronal excitability^[^^77^^,^^78^^]^. At the same time, injection of DHEA-S, a negative allosteric modulator of the GABA-A receptor, can increase the release of acetylcholine, which is a neurotransmitter closely associated with memory function in hippocampus^[^^79^^]^. Therefore, higher concentration of testosterone by acting as a non-selective sigma antagonist leads to lower NMDA receptor function. Therefore, the release of acetylcholine in hippocampus increased in the presence of steroid hormones^[^^80^^-^^82^^]^. 

In aged men, a positive correlation has been found for cognition process and free testosterone levels^[^^83^^]^. Moreover, according to a wide range population of men and women (35-90 years old), free testosterone concentration indicated a sex-specific behavior and impact on cognitive functions, including visuospatial ability, episodic learning, semantic memory, and verbal fluency^[^^65^^]^, while testosterone concentration represented positive trends with visuospatial learning in women. Nonetheless, attempts to find a connection between verbal fluency, semantic memory, and testosterone levels have been failed^[^^84^^,^^85^^]^. On the other hand, testosterone rate in women is believed to be positively or negatively related to episodic memory based on previous studies^[^^84^^,^^85^^]^, which is contradictory. 

**Fig. 3:**
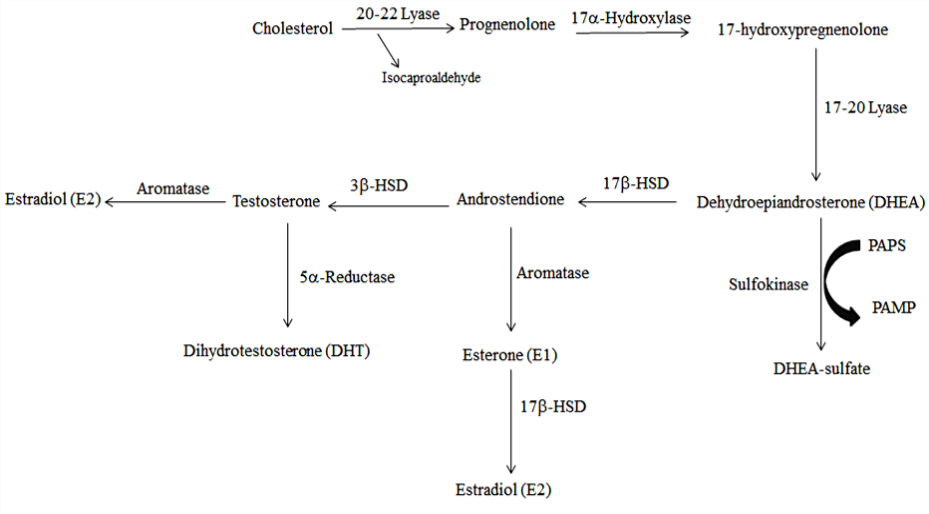
Biosynthesis of HSD and DHEA-S in the body

Conversion of testosterone to DHT via 5α-reductase, as well as to non-aromatic metabolite like 5α-androstane-17β-diol (3α-diol), by 3α-HSD has also been under discussion. Research has proposed that cognitive functions are improved in gonadectomized rats, which were systemically administrated by 3α-diol^[^^86^^]^. Additionally, a complementary study was conducted to observe the intrahippocampal administration effect of 3α-diol and indomethacin (as a 3α-HSD inhibitor which blocks testosterone and DHT conversion to 3α-diol) on spatial cognition. According to the results, it was concluded that simultaneous injection of 3α-diol, and indomethacin did not alter impairment influence of indomethacin or 3α-diol alone in Morris water maze taskl^[^^87^^]^. DHT treatment of rats also demonstrated the efficacy of DHT in reduction of escape latency and traveled distance in the Morris water maze, which proposed that testosterone metabolites may have significant impacts on memory functions^[^^88^^]^. Testosterone influence on the right and left hippocampus was also studied recently^[^^89^^]^. Notable reduction of the mentioned hormone was only detected in right hippocampus, while a low percentage of right hippocampus testosterone led to its conversion to other metabolites^[^^89^^]^. Furthermore, regarding the effect of testosterone on memory process, scientists observed that the number of astrocytes in the CA1 region of rats enhanced by memory impairment induction via testosterone^[^^90^^]^ (Fig. 3).

DHEA and DHEA-S have been proposed to be responsible for the acceleration of age-related physical and memory processes^[^^91^^]^. Inconsistent results have been obtained that indicate an inverse trend of DHEA-S concentration with aging-associated men’s and women’s memory impairment^[^^92^^]^. However, contradictory findings have suggested that DHEA-S reduction may not be attributed to cognitive functions^[^^93^^]^. Although DHEA and DHEA-S have been shown to improve the aging rodents’ memory functions^[^^94^^,^^95^^]^, based on another piece of evidence, no significant alteration was detected in spatial cognition of mice treated with DHEA-S^[^^96^^]^. 

The neuropsychiatric and cognitive influences of DHEA and DHEA-S are resulted from GABA, NMDA, and σ-receptor potentiation effects. The mentioned hormones enhance regional serotonin and dopamine function in brain, hippocampal primed burst potentiation and cholinergic function, anti-glucocorticoid activity, inhibition of proinflammatory factors production, and bioavailability of insulin-like growth factor I^[^^97^^-^^102^^]^. These results demonstrated the effect of DHEA-S in global cognition, working memory, attention, and verbal fluency. The difference between the effect of DHA and DHEA-S is probably related to the dose as well as the age of the patients. However, the safety of this treatment is controversial, and the risk of side effects may increase at higher doses^[^^103^^,^^104^^]^.

The functional role of testosterone and estrogen has formerly been distinguished by scientists. It was recommended that the defect in spatial memory process was associated with testosterone and estrogen, while anastrozole (an aromatase inhibitor) improved the cognitive functions^[^^105^^]^. In addition, owing to the significant role of estrogens in memory process, modulation of cognitive functions via inhibitors of ER (comprising of ERα and ERβ isoforms) was observed using ERα agonist PPT and ER antagonists flutamide and TAX. Based on the findings, the microinjection of PPT and TAX simultaneously in hippocampus CA1 region of male rats leads to the spatial cognition impairment, which may stand for the fact that there may be substitutional estrogenic mechanism for the regulation of memory processes^[^^106^^]^. The sensitivity to transcription and translation inhibitors (actinomycin D, cycloheximide, and anisomycin) is also characterized as the androgens genomic importance. Thus, research has been performed to observe anisomycin influence on genomic functions of testosterone. The simultaneous administration of testosterone and anisomycin have been indicated to increase the spatial cognition improvement^[^^74^^]^. 

**Protein kinases**

More than 500 protein kinases have been identified in humans^[^^107^^]^. Evolutionary studies have resulted in their classification based upon catalytic domains into seven major groups: tyrosine kinase, tyrosine kinase-like, homologues of yeast Sterile 7, 11, and 20 kinases, CDK, MAPK, GSK3, and CDK2-like kinase that lately they were named as CMGC, casein kinase 1, CaMK, and AGC kinase groups, including the protein kinase A, G, and C families^ [^^107^^]^. AGC kinase cluster is comprised of cAMP-dependent PKA, cGMP-dependent PKG, and PKC^[^^108^^,^^109^^]^. AGC kinase cluster affects various health issues including cancer, metabolic disorders, cardiovascular diseases, immunological disorders, muscular dystrophies, and neurological disorders^[^^110^^-^^114^^]^.

Protein kinases alter their target proteins' functions through the phosphorylation. In fact, protein kinases serve as essential factors regulating intracellular signaling pathways pertaining to cell growth, differentiation, development, functions, and death^[^^115^^]^. The effect of protein kinases on AD has been under discussion. Besides, PDE superfamily is located in brain^[^^116^^]^. Studies have suggested that PDE inhibitors may have significant impacts on spatial cognition and cholinergic activity in hippocampus^[^^117^^,^^118^^]^. Until now, little is known about the function of these types of esterases. Indeed, cAMP and cGMP pathways incorporate in AD, while it is believed that their concentration is increased by PDE inhibitors. Hence, Hosseini-Sharifabad *et al*.^[^^119^^]^ focused on PDE/protein kinases A and G relationship influence on cholinergic activity and memory impairment. According to their findings, they recommended that the PDE inhibitor promotion of cAMP/PKA- and cGMP/PKG-mediated pathways activities could increase the spatial memory in hippocampus, while chronic enhancement of cholinergic activity was not confirmed. Additionally, PKAII impact on spatial learning has been observed lately. The published data suggest that PKAII inhibition could affect the spatial memory. Nonetheless, when a PKAII inhibitor, like H-89, was co-administered with testosterone, negative correlation in memory process was detected^[^^120^^]^. Furthermore, the role of PKAII in cholinergic gene expression modulation has been studied, which confirms that PKAII acts as an important agent in spatial cognition retention in male rats^[^^121^^]^. However, the widely known protein kinase responsible for memory processes is PKC, for which several investigations have been conducted to elucidate its detailed functions in various signaling pathways.

**PKC classification and its isoforms**

The serine/threonine PKC family consists of 12 various isoforms, which are known to be involved in brain signaling pathways and related regulations, i.e. cell growth, differentiation, apoptosis, transformation, tumorigenicity, synaptic function, behavior, and cognition^[^^9^^,^^122^^]^. Typically, three different subgroups are characterized as PKC isozymes: classical PKC of α, βI, βII, and γ, novel PKC of δ, ε, η, θ, and μ, and atypical PKC of ζ, ι, and λ, including PKMζ^[^^9^^]^. However, PKCα, PKCβΙ, PKCβΙΙ, PKCγ, PKCδ, PKCε, PKCθ, and PKCη are determined as the eight homologous isozymes. The intact PKC is believed to be activated by DAG, while subsequently, it interacts with the tumor-promoter phorbol ester in the membranes^[^^123^^,^^124^^]^. Regarding the memory process, PKC isozymes, particularly PKCα, PKCγ, PKCε, and PKCζ, share essential roles in signaling pathways, which gained researchers' interest in the memory kinases as the possible therapeutics for cognition disorders, e.g. AD^[^^9^^]^.

PKC is composed of two major domains: the regulatory domain (consisting of C1 and C2) and the catalytic domain (more conserved than the regulatory domain), including C3, the ATP-binding domain, and C4, the protein substrate-binding domain linked by an isozyme-specific variable region^[^^9^^,^^123^^]^. 

**Role of PKC in cell signaling and AD**

Gene expression studies of PKC isozymes have indicated that most isoforms are present during development period in all tissues. These isoforms still play opposing roles in various signaling states in the same cell. Indeed, phorbol ester is determined as an ineffective activator of PKC rather than DAG, which is a transient inducer^[^^123^^]^. The PKC critical role in several disorders, including diabetes^[^^125^^]^, cancer^[^^126^^]^, ischemic heart disease^[^^127^^]^, heart failure^[^^126^^]^, autoimmune diseases^[^^128^^]^, PD^[^^129^^,^^130^^]^, AD^[^^131^^]^, bipolar disorder^[^^132^^,^^133^^]^, psoriasis^[^^134^^]^, stroke^[^^135^^]^, dementia^[^^8^^]^, and pain^[^^136^^]^, has been discussed so far. 

In β-amyloid cascade, APP cleavage by α-secretase or β-secretase is regulated by PKC enzyme exclusively, which is deficient in AD patients. Specifically, α-secretase induction is modulated directly by α and ε isozymes or indirectly through ERK1/2 activation by PKC, or simultaneously through both ways^[^^122^^]^. Through α-secretase activity of APP processing, phosphorylation is increased via PKC induction. In fact, translocation of PKCα and PKCε from cytosol to the membrane and Golgi-like structures occurs by phorbol ester stimulation of α-secretase. As a consequence, the PKC phosphorylates MAPKs ERK1/2, as well as tumor necrosis factor-α converting enzymes family. It also acts as an α-secretase activity enhancer. Although statins (cholesterol-lowering drugs) are found to be α-secretase substitute activators, which could also increase sAPP release by α-secretase, PKC or ERK1/2 do not interfere with the process. From another point of view, β-amyloid oligomers have the potential role to inactivate PKC through Aβ_28-30_ residues^[^^137^^]^. Furthermore, Aβ_1-40_ has degradative impact on PKC α and γ isozymes in normal and AD patient individuals^[^^138^^]^. Reduction in the phosphorylation of soluble brain proteins through PKC has also been reported to happen at increased concentration levels of Aβ_1-40_^[^^139^^]^. Additionally, experimental works have confirmed that PKCε presents degradative effects on Aβ levels *in vitro* and *in vivo*^[^^140^^,^^141^^]^. Tau proteins, along with β-amyloid peptides that stand for memory process regulations, show the ability to bind to MT structures through serine/threonine-directed phosphorylation. Indeed, the tau-MT binding is induced by tau dephosphorylation, while their dissociation is promoted by tau phosphorylation. The GSK3, CDK5, the MARK, and ERK1/2 relation in balance are accounted for the modulation of tau phosphorylation^[^^142^^]^. In particular, Aβ_1-42_ promotes the activation of ERK1/2, which subsequently results in tau hyperphosphorylation, and eventually neuro-degeneration. However, GSK-3β plays a key role in tau phosphorylation^[^^143^^]^.

PKC has potential impact on the inhibition of GSK-3β through the direct process of tau phosphorylation and neurofibrillary tangle reduction^[^^144^^]^. GSK-3β could also be inhibited indirectly by the lower production of Aβ_1-42_ via PKC^[^^145^^]^. The symptoms of PKC defect include memory loss and reduced PKC and α-secretase activities, leading to the higher levels of Aβ peptides, and as a consequence, the formation of amyloid plaques. Moreover, decline in the GSK-3β inhibition via the decreased activity of PKC results in hyperphosphorylation of tau proteins, and ultimately inflammation. Additionally, the reduced PKC activity could be attributed to aging, which is an important risk factor^[^^146^^]^. Thus, PKC may serve as a candidate therapeutic agent or a target drug owing to its significant role in memory process.

**PKC and androgens**

**Fig. 4:**
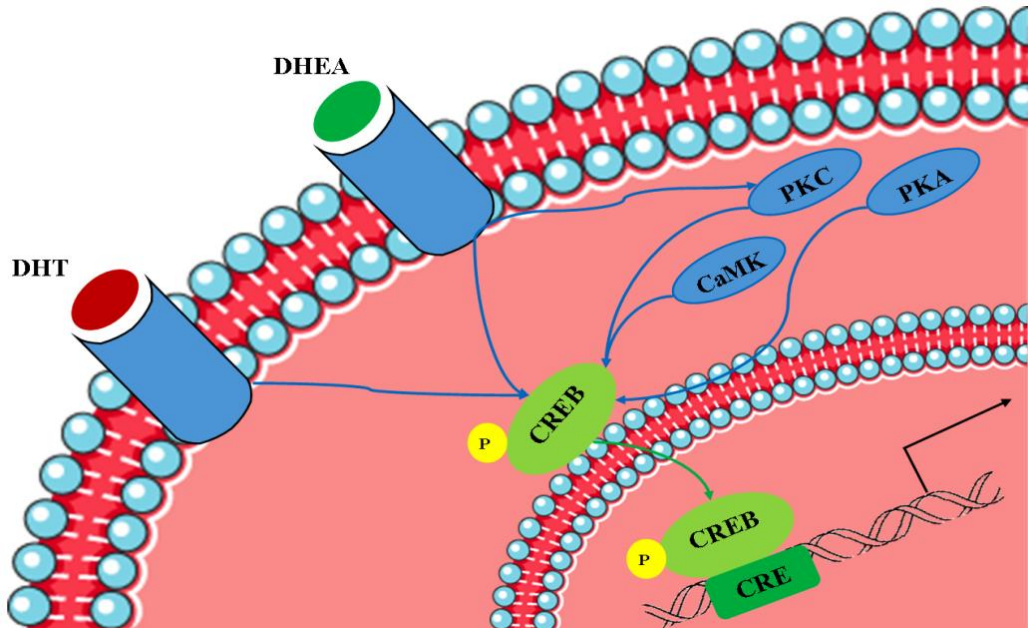
CREB signaling pathway. Several signaling pathways, including those involving PKA, PKC, DHT, and DHEA, have been associated with the regulation of *de novo *protein synthesis in the context of synaptic plasticity, converging on the phosphorylation of CREB at Ser133 residue to repair cognition dysfunction. PKA, protein kinase A; DHT, dehydrotestosterone; DHEA, dhedroepiandrosterone; CRE, cAMP response elements. The following binding DHT and DHEA to their receptors in the cytoplasmic membrane, several enzymes like PKC, PKA and CaMK are activated. On the other hand, the mentioned hormones activates CREB protein directly using phopsphorylation on Ser133. Activated CREB protein passes through the nucleus membrane and is bonded to its receptor to occur the gene expression

Androgen deficiency is a major risk factor in aged men. Among the key impacts of aging on functional and behavioral processes is reduction in the potency of immune response, which leads to decreased innate and adaptive immunity responses^[^^147^^]^. PKC signaling pathways are associated with lower expression levels of RACK1, which is a kinase and a membrane receptor scaffold protein. Deficiency in PKC can result in reduced functional immune responses related to aging^[^^148^^]^. In fact, active conformational stabilization of PKCβII is dependent on its attachment to RACK1, while its translocation is induced by specific PKCβII substrates, which are critical for immune cell activation, proliferation, differentiation, and survival^[^^149^^,^^150^^]^. According to earlier investigations, memory impairment and cognitive dysfunctions are affected by the mentioned PKC signaling deficiency^[^^151^^,^^152^^]^. Indeed, age-dependent decrease of DHEA has been found to be correlated with the reduced expression levels of RACK1. Moreover, *in vitro* and *in vivo *findings declare that DHEA administration in aging cells of animals and humans may lead to RACK1 recovery^[^^153^^]^. Interestingly, cortisol levels remain to be unaltered during lifetime, which results in the total increase of cortisol: DHEA ratio^[^^153^^]^. Knowing the fact that cortisol demonstrates a negative correlation with RACK1, then, DHEA defects can cause the prevention of cortisol activity and, therefore, the induction of RACK1 expression. However, experiments have revealed that pretreatment by DHEA promotes the RACK1 counterbalance, which had formerly been decreased by cortisol regulatory effect^[154]^. From another point of view, CREB is a potent signaling molecule, which is thought to be associated with androgen neuroprotective functions in MAPK/ERK pathway^[^^155^^-^^158^^]^. As a matter of fact, down-regulation of MAPK/ERK signaling pathway is in part attributed to CREB, while PI3K/Akt^[^^159^^]^, PKA^[^^160^^]^, CAMK IV^[^^161^^]^, and PKC^[^^162^^]^ are responsible for regulating CREB activity. Multiple neurotrophic and neuroprotective impacts are believed to be mediated by active CREB function in neurons^[^^163^^,^^164^^]^. Although androgens are key activators of CREB pathway in non-neural cells^[^^165^^-^^167^^]^, there is little information about the androgens role in CREB signaling activity in neurons. Study on androgens possible role in the induction of CREB activities in primary hippocampal neuron cultures have suggested that CREB molecules show enhanced phosphorylation due to intracellular AR activation^[^^168^^]^. Interestingly, androgen-dependent phosphorylation of CREB is not prevented by the upstream CREB signaling pathways MAPK/ERK, PI3K/Akt, PKA, or CaMKIV pharmacological inhibition. However, PKC deficit or its pharmacological inhibition resulted in CREB phosphorylation blockage, which recommends that CREB signaling pathway in neurons is related to AR and PKC^[^^168^^]^ (Fig. 4).

In PD, as a memory disorder, dopamine neurons are defective^[^^169^^]^, which is mediated by oxidative stress^[^^170^^]^, leading to cellular apoptosis^[^^171^^,^^172^^]^. According to studies, testosterone is related to the development of PD by the enhancement of apoptotic pathway^[^^173^^,^^174^^]^; nonetheless, the related death of dopamine neurons and testosterone correlation have not well been established. The apoptosis pathway is determined to be regulated by caspase-3^[^^175^^,^^176^^]^, when cell death occurs following PKC processing via caspase-3 activation. In PD as a memory disorder, dopamine neurons are defective^[^^169^^]^, which is mediated by oxidative stress^[^^170^^]^ leading to cellular apoptosis^[^^171^^,^^172^^]^. According to studies, testosterone is related to the development of PD by the x, when cell death occurs following PKC processing via caspase-3 activation^[^^177^^]^. Consistent results have also declared the importance of PKC in oxidative stress and testosterone functions^[^^178^^,179]^. Based upon the study of Cunningham *et al.*^ [^^180^^]^, promotion of apoptosis showed to be occurred following PKC-dependent activation of caspase-3, via testosterone and DHT in dopaminergic neuronal cells. Although few experiments have discussed the significance of androgens in PKC functional behavior, the key fundamental impacts need to be identified. The detailed correlation of androgens with PKC signaling pathways has slightly been known, but further investigations remain to be performed. In this sense the study of androgens/PKC association may lead to the discovery of potent therapeutic agents. 

**Relationship between PKC, androgens, and** **AD**

**Fig. 5:**
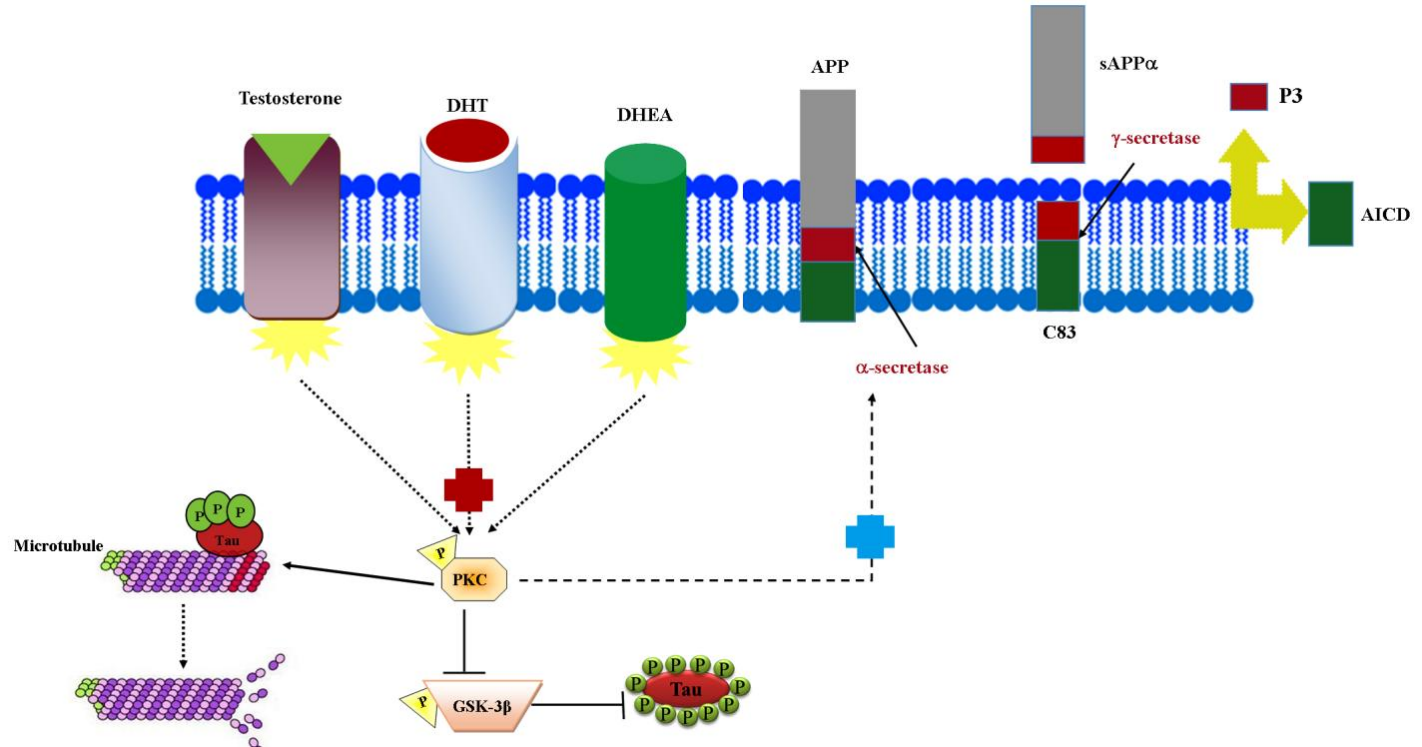
Effect of androgens on induction of non-amyloidogenic pathway of AD. AICD, APP intracellular domain. P stands for phosphorylated protein, and (**+)** in the image describes the activated effect on the group. Hyperphosphorylated tau containing several phosphate groups attached to the tau protein has been shown. PKC leads to activate the polymerization and depolymerization of MT protein via hyperphosphorylated tau in the normal conditions; therefore, it can help the electron transfer in the nervous systems and create action/potential in the synaptic ends

Genetic and environmental factors result in higher neural accumulation of Aβ in brain^[^^181^^]^, which is a critical factor in AD pathogenesis. Previous studies have demonstrated that decrease in endogenous androgens significantly enhances Aβ accumulation in brain. Thus, it could be concluded that androgens play important roles as the regulators of neural Aβ levels. However, loss of this function can promote AD pathogenesis^[^^167^^]^. Testosterone and DHT can also change APP processing and decrease Aβ levels in cultured cells by a mechanism that involves the activation of AR-dependent pathways, indirect activation of estrogen pathways via aromatization to estradiol, and modulation of gonadotropin actions via regulation of the hypothalamic–pituitary–gonadal axis^[^^182^^]^. The activation of AR is related to several protein kinases such as MAPK and/or PKC^[^^183^^,^^184^^]^. DHEA, DHEA-S, and testosterone also decline with age in brain tissue in men, which can give a rise to the working memory impairment^[^^185^^,^^186^^]^. PKC is crucial for hippocampal memory formation. Activated PKC can affect signaling pathway in the presence of the mentioned androgens in AD. The special isoforms of PKC like PKC and PKCε can work directly on -secretase; hence, they can trigger A degradation in the brains of PKCε transgenic mice that express amyloidogenic variants of human APP^[^^140^^]^. PKC both 

directly phosphorylates tau and indirectly causes the dephosphorylation of tau by phosphorylating and inactivating GSK-3β^[^^62^^]^. Tau protein has been recognized as a major neuronal MAP, which promotes MT polymerization and stabilizes MT polymer structure^[^^187^^]^. MTs are composed of two subunits, α- and β-tubulin, with high negative charges at the C-terminal end^[^^188^^]^. The interaction between MT and tau is regulated through phosphorylation and dephosphorylation on tau protein by several enzymes such as kinases like PKC, GSK3β, and phosphatases^[^^189^^,^^190^^]^. GSK3β is the primary protein kinase that regulates tau phosphorylation in brain^[^^191^^]^. GSk3β regulates several signaling pathways in tau pathology and plays an inhibitory role in AD pathophysiology and cell division process. PKC activation lessens tau hyperphosphorylation by inhibiting GSK3β; the inhibition of GSK3β is transpired by phosphorylation in serine 9^[^^144^^]^. Reducing Aβ1-42 production using PKCε, the most important enzyme involving in AD, can lead to the inhibition of GSK-3β and consequently, the reduction of tau phosphorylation and neurofibrillary tangles. PKC-α partakes in tau phosphorylation, which is controlled by the intracellular level of cAMP^[^^192^^]^. The alterations in PKCγ contribute to deficits in hippocampal-mediated memory in the aged individuals^[^^193^^]^ (Fig. 5). 

To conclude, we have focused on learning and memory process, in which androgens play significant regulatory roles. Overall, androgens expression levels are reduced throughout the lifetime, and their deficiency can lead to a number of behavioral and functional alterations, including cognition and memory impairment. Additionally, PKC modulates several signaling pathways dependent on memory process. In particular, PKCα and PKCε directly regulate α-secretase induction, while indirectly modulate α-secretase through ERK1/2 activation by PKC. Tau proteins phosphorylation is also induced via its binding to PK; therefore, phosphorylated tau is dissociated from MT, while the formation of Aβ_1-42_ is promoted by GSK3, CDK5, MARK, and ERK1/2. However, GSK-3β could be inhibited directly or indirectly through PKC pathways or Aβ_1-42_ lower production via PKC. Although androgens role in cognition represents contradictory results, DHEA-S has been found to be negatively correlated with phosphorylated tau protein concentration and Aβ oligomers levels. Furthermore, testosterone treatment declines the tau hyper-phosphorylation, while androgens seem to promote non-amyloidogenic APP processing. Besides, deficient PKC signaling pathways are associated with lower expression rate of RACK1 scaffold protein, which leads to age-dependent decrease of immune response function. In addition, DHEA reduction is correlated with cortisol (the negative regulator of RACK1), DHEA ratio increase and RACK1 decline, for which research has suggested that the DHEA injection may restore RACK1 expression levels. PKC regulates CREB activity related to MAPK/ERK signaling pathway in hippocampal neurons. Indeed, CREB activity and its increased phosphorylation are suggested to be AR- and PKC-dependent. Neurons death in PD has also been attributed to testosterone activity and its correlation with PKC, which is processed by caspase-3 activation. Studies have evidenced the fundamental correlation of androgens and PKC, which may eventually serve as a potential treatment for memory impairment. Further experiments are recommended to reach accurate and consistent results.

## CONFLICT OF INTEREST.

 None declared.
